# Effect of Copper Ion Sterilization on Bacterial Community in a Freshwater Recirculating Aquaculture System

**DOI:** 10.1007/s00284-021-02707-2

**Published:** 2022-01-04

**Authors:** Jianjun Shan, Xiaoqing Tian, Chongwu Guan, Chenglin Zhang, Yulei Zhang, Shi Chen

**Affiliations:** 1 Fishery Machinery and Instrument Research Institute, Chinese Academy of Fishery Sciences, Ministry of Agriculture and Rural of the People’s Republic of China, Shanghai, 200092 People’s Republic of China; 2 East China Sea Fisheries Research Institute, Chinese Academy of Fishery Science, Shanghai, 200090 People’s Republic of China; 3 Shanghai Ocean University, Shanghai, 201306 People’s Republic of China

## Abstract

The study aimed to evaluate the safety of copper ion sterilization based on copper ion residues in zebrafish (*Brachydanio rerio*), as well as bacterial community structure and diversity in recirculating aquaculture systems (RASs). The copper ion content was determined using national food safety standard GB 5009.13-2017. Bacterial community structures and alpha and beta diversity indexes were examined using the 16S rRNA gene sequences produced by Illumina HiSeq sequencing. The results revealed no significant copper ion enrichment in *B. rerio* when the copper ion concentration was 0.15 mg/L. The relative abundances of *Erythrobacter*, nitrite bacteria, and *Flavanobacteria* were clearly higher in the treatment group than in the control and differences in bacterial species richness and diversity were obvious. In addition, there was no sharp decrease in the microflora at the outflow of the copper ion generator. In conjunction with the changes in ammonia nitrogen, nitrate, and nitrite concentrations during the experiment, the results indicated that there were no significant effects on the purification efficacy of the biological filter, but the abundances of beneficial bacteria increased significantly. This is of great relevance in order to understand the response of bacterial communities affected by changing environmental conditions, such as copper ion sterilization.

**Supplementary Information:**

The online version contains supplementary material available at 10.1007/s00284-021-02707-2.

## Introduction

As the aquaculture industry has developed, the number of farmed aquatic species has increased [1]. In addition, improved living standards have allowed consumers to focus on comprehensive nutrition and to demand increasingly higher safety standards in aquaculture production [2]. The health of the aquaculture environment is closely related to the microbial community structure in the water, as well as the levels of nutrients [3]. Microbial community structure, which reflects the biochemical reactions in the ecosystem, can be used as an indicator of the ecological health of the aquaculture environment [4]. Because aquaculture species must be fed constantly, native microorganisms cannot decompose organic matter rapidly enough to prevent water quality deterioration and the growth of harmful microorganisms [5]. The excess undecomposed feed not only pollutes the aquatic environment but also inhibits the respiration of other aquatic animals, possibly even leading to death [6]. High levels of organic matter pollution may also be toxic to humans [7]. However, beneficial microorganisms may play a role in water purification and may serve as food for aquatic organisms [8, 9]. Therefore, beneficial and harmful aquatic bacteria should be analyzed effectively to ensure the health of aquaculture environments.

Sterilization technology can effectively mitigate the conflict between ecological benefits and economic interests, supporting sustainable development [10]. Copper ion sterilization is an electrochemical sterilization method and copper ions are also one of the most common heavy metal contaminants in water [7]. The author's previous study found that the concentrations of nitrite, nitrate, and total ammonia nitrogen (TAN) and the total number of bacteria remained in a stable state when the concentration of Cu^2+^ is 0.1–0.2 mg/L and the breeding temperature was (26 ± 2) °C. These conditions were beneficial for the growth of zebrafish (*Brachydanio rerio*) [7]. However, it is unclear whether *B. rerio* retains copper ions over the long term in aquaculture.

This study thoroughly discusses the safety of the copper ion sterilization process in aquaculture water and provides a theoretical reference for the aquaculture industry with respect to copper ion residue and the microbiome.

## Materials and Methods

The experimental system included a storage tank, a biochemical cotton filter, a moving bed biofilm reactor (MBBR), and a copper ion generator. The filter media parameters were as follows: K5, Ф25*4 mm, 64 holes, the specific surface area is 800 m^2^/m^3^, the bulk number was 2.1*10^5^ per m^3^, the bulk weight was 125 kg/m^3^, and the void ratio was 85%. The copper ion generator used was YLD-TLZ-25, Jiangsu Yilida Water Treatment Systems Co., Ltd., China.

Two recirculating aquaculture systems (RASs) for *B. rerio* were constructed. *B. rerio* were acclimated for seven days in an RAS before the experiment began. Fifty healthy *B. rerio* with an average length 2.5–3.0 cm and a weight of 0.30 ± 0.05 g were selected.

The fish were divided into a control group and a treatment group. The control and treatment groups were exposed to 0 mg/L and 0.15 mg/L copper ions, respectively. Daily management operations included regular feeding and system sewage treatment. The copper ion concentration was tested three times per week, while TAN, nitrite, nitrate, dissolved oxygen (DO), temperature (T), and pH were tested daily. Samples of the water and filter material were collected at the end of the experiment. TAN, nitrate, and nitrite concentrations were determined using Nessler’s reagent spectrophotometry, zinc–cadmium reduction, and diazotization coupling, respectively. This experiment was performed at the Fisheries Equipment and Engineering research base of the Fishery Machinery and Instrument Research Institute, Chinese Academy of Fisheries Sciences, China in July 2018. The experiment lasted one month.

The RAS water samples (100 mL each) and the filter media samples from MBBR (100 g each) were collected in sterile tubes separately and transferred to the laboratory. Samples were collected in triplicate. The filter was washed with sterile fresh water and shaken vigorously to dissolve the biofilm on the carrier. Then, the above solutions were filtered onto 0.22 μm filters and frozen at − 80 °C. Ten *B. rerio* each in the treatment group and control group were randomly selected to measure the concentration of copper ion residues in *B. rerio* tissue.

In the treatment group, the biological filter sample was marked S1, the water sample was marked S, and the water sample from the outlet of the copper ion generator exit was marked O. In the control group, the biological filter sample was marked N1, and the water sample was marked N.

To maximize read depth for a temporal study of the bacterial communities in the RAS, we used the Illumina HiSeq platform and targeted the V3–V4 region of the 16S rRNA gene. The V3–V4 region of the bacterial 16S rRNA gene was amplified using the forward and reverse universal primers 338F (5′-ACTCCTACGGGAGGCAGCA-3′) and 806R (5′-GGACTACHVGGGTTCTAAT-3′), respectively. The amplicon mixture was applied to a HiSeq 2500 MiSeq Genome Sequencer (Illumina, San Diego, CA, USA). Bacterial community composition and biodiversity were determined by Shanghai Majorbio Bio-Pharm Technology Co., Ltd. (Shanghai, China).

### Data Analysis

The extracted high-quality sequences were aligned using PyNAST and UCLUST. The unique sequences were classified into operational taxonomic units (OTUs) at a threshold of 97% identity using UCLUST. Chimera Slayer was used to remove potential chimeric sequences from the set of representative OTUs. MOTHUR was used for data analysis. The data were analyzed on the free online platform of Majorbio Cloud Platform (www.majorbio.com). Statistical analyses were performed using SPSS V.17.0 [11]. The mean (M) and standard deviation (SD) were used to explore the relationship between specific explanatory variables and outcome variables for water quality results and concentration of copper ions in *B. rerio* after exposure to 0.15 mg/L copper ions.

### NCBI Sequence Accession Numbers

Bacterial V3–V4 16S rRNA gene sequences generated in this study are available from the NCBI SRA (SRP297447).

## Results

### RAS Water Quality Parameters

**Fig. 1:**
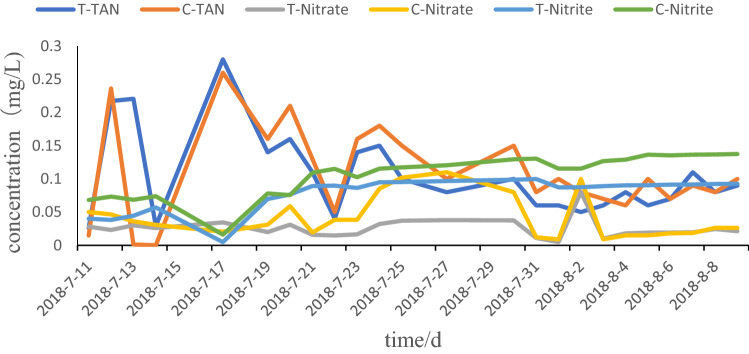
The results of water quality. *In this figure, the treatment groups were marked T- and the control groups were marked C-.The concentrations of TAN, nitrate, and nitrite were 0.13 ± 0.08 mg/l, 0.02 ± 0.01 mg/l, and 0.02 ± 0.01 mg/l in the test group and 0.14 ± 0.07 mg/l, 0.05 ± 0.04 mg/l, and 0.03 ± 0.02 mg/l in the control group

The concentrations of TAN, nitrate, and nitrite in the test group were 0.04–0.35 mg/L, 0.04–0.01 mg/L, and 0.03–0.01 mg/L, respectively, with averages of 0.13 ± 0.08 mg/L, 0.02 ± 0.01 mg/L, and 0.02 ± 0.01 mg/L, respectively. In the control group, the concentrations of TAN, nitrate, and nitrite were 0.05–0.26 mg/L, 0.18–0.01 mg/L, and 0.08–0.02 mg/L, respectively, with averages of 0.14 ± 0.07 mg/L, 0.05 ± 0.04 mg/L, and 0.03 ± 0.02 mg/L, respectively (Fig. 1).

### Residual Copper Ions in *B. rerio*

*B. rerio* were sampled one day after the last feeding. Selecting some whole fish sample using graphite furnace atomic absorption spectrometry (NHC 2017) detect the copper ion content, respectively. The average values of the cupric ion concentrations in the treatment and control groups were both 16.3 mg/kg.

### Influence of Copper Ions on the Microbiology of the RAS

Based on homologous sequence alignments and clustering, using the information extracted from the RDP and BLAST databases, the OTUs were taxonomically identified to the lowest level possible. The Shannon diversity curves for those samples reached the saturation phase, indicating that the majority of the bacterial phylotypes in the sample had been identified (Supp. Fig. I).

Alpha-diversity data for the samples generated by high-throughput sequencing are shown in Supp. Table1, including the Shannon, Simpson, Chao, and Coverage indexes. The estimated sample coverage (Good’s coverage) was about 99%, which indicated that sequencing accuracy and reprehensibility was acceptable. No significant difference in estimated OTU richness (Chao) was observed between the two groups.

**Fig. 2:**
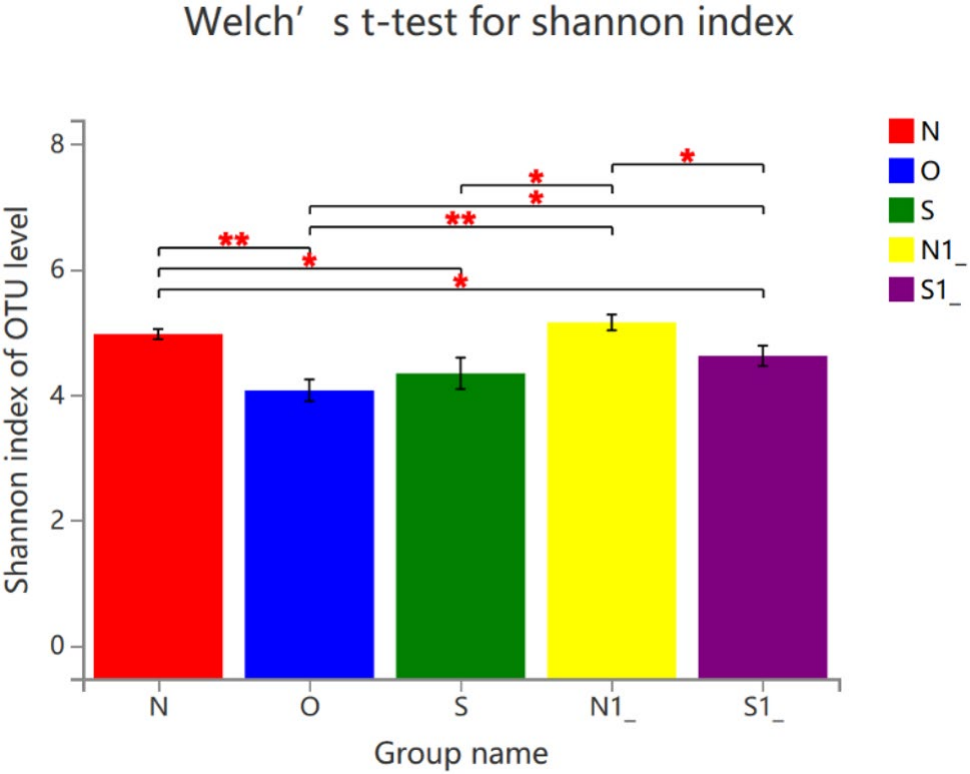
Microbial diversity among groups, as measured by Shannon index. *Data are expressed as mean ± standard deviation (S.D.) for each group (*n* = 3). *P < 0.05, **P < 0.01, ***P < 0.001. Error bars show the standard error of mean (S.E.M.), and the *P*-values are from two-tailed Student’s *t* test

However, significant differences in estimations of community diversity between the two groups were identified, including the Shannon index at 4.62 ± 0.16 in the treatment group vs. 5.15 ± 0.13 in the control, with P = 0.01 and 4.34 ± 0.25 in the treatment group vs. 4.96 ± 0.08 in the control, with P = 0.04 for the filter and water samples, respectively, indicating that microbiota diversity was greater in the treatment groups. In addition, there were no significant differences in estimations of community diversity between the S and the O in the treatment groups (Shannon 4.34 ± 0.25 vs. 4.07 ± 0.17, *P* = 0.2) (Supp. Tab.1 and Fig. 2).

**Fig. 3:**
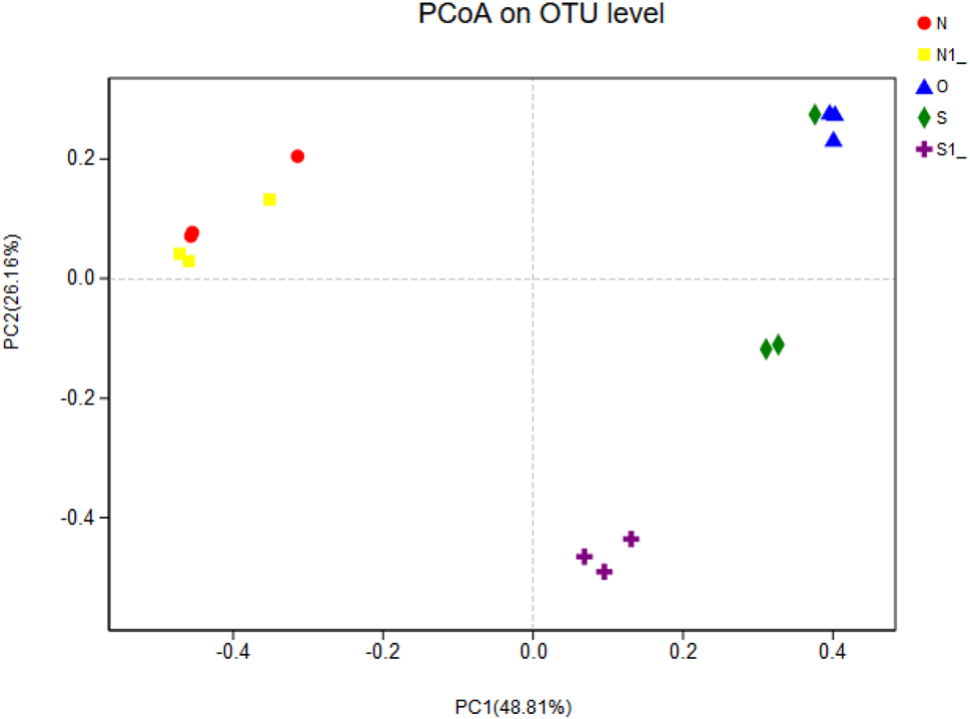
Principal coordinate analysis plots. *showing the microbiota in the treatment groups and the control groups (*n* = 3 per group). Principal co-ordinates analysis (PCoA) of the sequencing data identified differences between the treatment-group clusters and the control-group clusters, with the following main principal component (PC) scores: PC1 was 48.81% and PC2 was 26.16%

Bacterial differences were further investigated. Principal co-ordinates analysis (PCoA) of the sequencing data identified differences between the treatment-group clusters and the control-group clusters, with the following main principal component (PC) scores: PC1 was 48.81% and PC2 was 26.16%. These PC scores demonstrated that the clustering pattern differed among groups (Fig. 3). N and N1 were in the same area and were distinct from S, S1, and O, which indicated that bacterial community structure differed between the treatment group and the control group. This suggested that copper ion sterilization had a significant effect in the RAC.

**Fig. 4:**
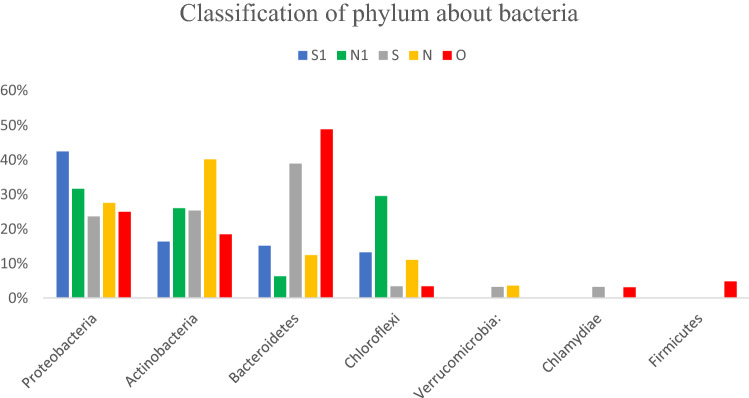
Analysis of Bacterial community composition in the filter and water samples on the phylum level. *The five most-abundant bacterial classes in the control group were Actinobacteria (20.04%), Gammaproteobacteria (17.88%), Alphaproteobacteria (14.82%), Caldilineae (8.14%), and Deltaproteobacteria (4.86%), while the five most-abundant bacterial classes in the treatment group were Alphaproteobacteria (26.65%), Actinobacteria (13.34%), Flavobacteria (11.59%), Gammaproteobacteria (9.0%), and Thermomicrobia (8.81%)

The species abundance of each sample was calculated at different taxonomic levels by community composition analysis. At the phylum level, the relative abundance of the Proteobacteria was high in filter material from both the control group and the treatment group, accounting for 31.6% and 42.4%, respectively (Fig. 4). The five most-abundant bacterial classes in the control group were Actinobacteria (20.04%), Gammaproteobacteria (17.88%), Alphaproteobacteria (14.82%), Caldilineae (8.14%), and Deltaproteobacteria (4.86%), while the five most-abundant bacterial classes in the treatment group were Alphaproteobacteria (26.65%), Actinobacteria (13.34%), Flavobacteria (11.59%), Gammaproteobacteria (9.0%), and Thermomicrobia (8.81%).

There were differences in the water samples between the treatment group and the control group. The dominant phylum in the control group was Actinomycetes, accounting for 40.10%, while the dominant phylum in the treatment group was Bacteroidetes, accounting for 38.90% in the S and 48.80% in the O (Fig. 4). However, the communities of low-abundance microbes were similar between the S and O in the treatment group, indicating that the aquatic environment was homogeneous.

**Fig. 5:**
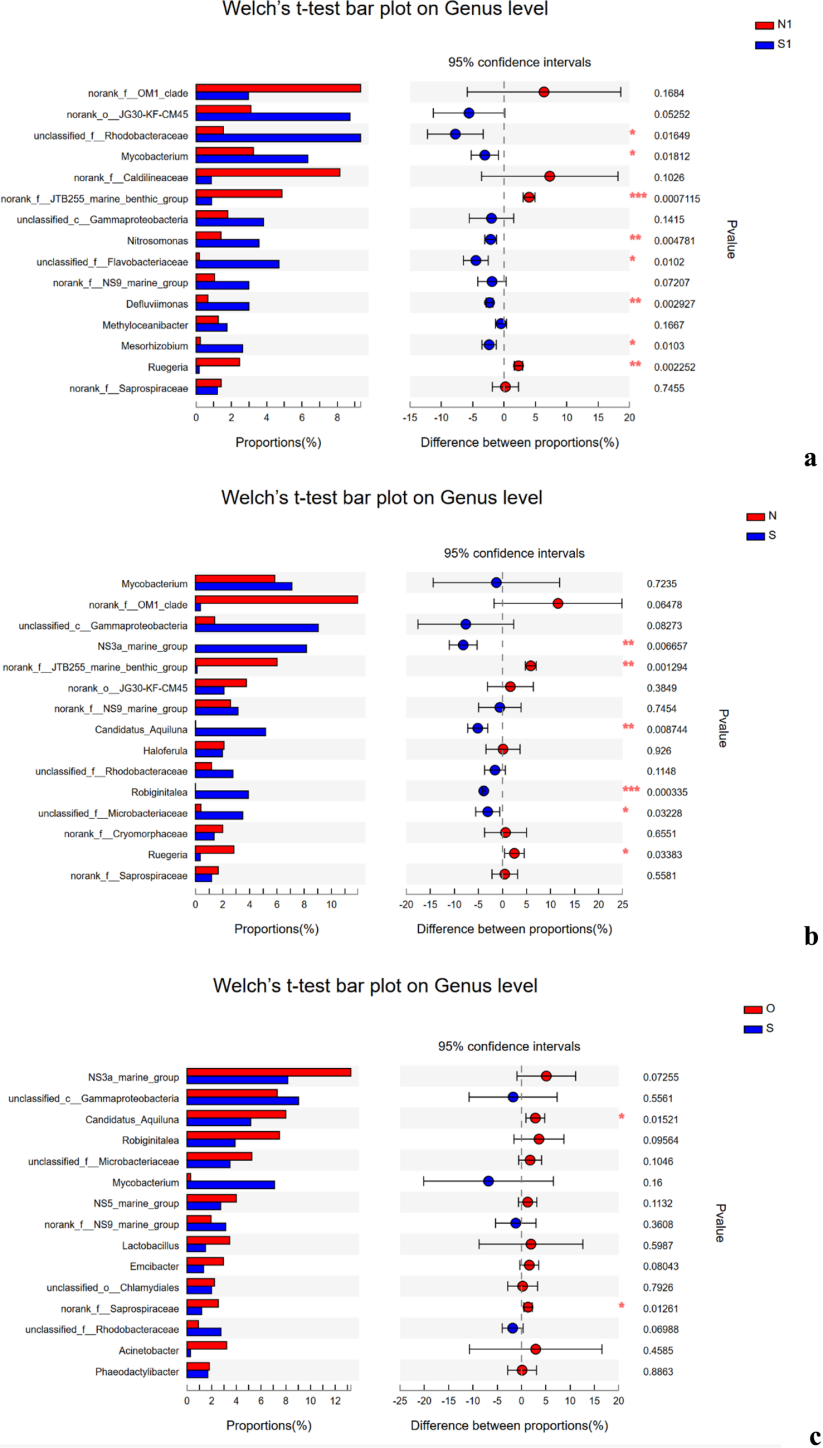
Significant differences in bacterial community composition between the treatment group and the control groups at the genus level. *Statistical analysis was performed using Welch’s *t* test (*n* = 3 per group). *P < 0.05, **P < 0.001; treatment group vs. control group

Species difference analysis revealed significant differences between groups by Welch’s *t* test. There were differences in the microflora genera between the treatment group and the control group. The genera that were significantly different in the filter included *Mycobacterium*, *Rhodobacteraceae*, *Defluviimonas*, *Flavobacterium*, *Nitrosomonas*, *Mesorhizobium*, and *Ruegeria* and the overwhelmingly dominant genus was *norank_f__JTB255_marine_benthic_group*. However, in water, *NS3a_marine_group*, *norank_f__JTB255_marine_benthic_group*, *Candidatus_Aquiluna*, *unclassified_f__Microbacteriaceae*, and *Ruegeria* were significantly different and the overwhelmingly dominant genus was *Robiginitalea*. Both *norank_f__JTB255_marine_benthic_group* and *Ruegeria* were more abundant in the control group than in the treatment group. In addition, there were significant differences in the abundance of the same strain between the water samples and filter materials (Fig. 5).

In the treatment group, only *Candidatus_Aquiluna* and *norank_f__Saprospiraceae* were significantly different between the S and O. In the control group, *norank_f__Caldilineaceae* and *norank_f__Saprospiraceae* were detected on the filter, while *norank_f__Saprospiraceae*, *Haloferula*, and *norank_f__Cryomorphaceae* were detected in the water (Fig. 5).

The relative abundances of *Erythrobacter*, nitrite bacteria*,* and *Flavanobacteria* were greater in the treatment group than in the control group. These microorganism were also most abundant in the sewage treatment and nitrogen removal systems in the biofilm reactor.

## Discussion

Various factors affect the survival of aquatic economic animals in RASs, including differences in system scale, water properties, and microbial community composition. Suitable water conditions are essential for the survival of aquatic economic animals, such as fish. In this experiment, there were no obvious changes in TAN, nitrate, and nitrite when the copper ions were 0.15 mg/L. The average values of the tested water properties did not reach harmful levels that would negatively impact aquaculture organisms [7]. In addition, there was no significant enrichment of copper ions in *B. rerio* when the copper ions were 0.15 mg/L.

Microbial ecological theory in aquaculture has the potential to extend RAS capabilities [12]. Further identifying the interactions between microorganisms and system design could alleviate concerns regarding the sustainability of aquaculture. The results of Bartelme et al. [13] studied the bacterial and archaeal community structure of a commercial-scale freshwater RAS raising *Perca flavescens* (yellow perch) and found that > 99.9% of the archaeal 16S rRNA gene sequences were classified to a single taxon associated with known ammonia-oxidizing archaea. Therefore, archaea will be focused on in a spatial study related to nitrification in the future.

It is likely true that each microbial community assemblage will be unique among RASs, i.e., each RAS has a unique “microbial fingerprint” [13]. The Shannon index, Simpson diversity index, Chao1, and observed species in each sample were used to evaluate species richness and diversity. The results indicated that bacterial species richness and community diversity varied distinctly among the samples. There were differences in the microflora genera between the treatment group and the control group, especially within *Rhodobacteraceae, Flavobacteriaceae, Haloferula*, and nitrite bacteria.

*Rhodobacteraceae unclassified* belongs to the family *Rhodobacteraceae*, in the Alphaproteobacteria, and is a purple non-sulfur bacteria [14]. Depending on extracellular enzymes, heterotrophic metabolic reactions can be carried out by utilizing a variety of organic carbon sources in aquaculture water [14]. In anaerobic or hypoxic conditions, *Rhodobacteraceae* effectively absorbs phosphate, reduces the phosphorus load in high-density aquaculture, and can play a role in reducing the chemical oxygen demand of water [15]. *Flavobacteriaceae* unclassified, which belongs to the *Flavobacteriaceae* (Bacteroidetes), is a facultative anaerobic bacterium. This bacterium performs dissimilatory nitrate reduction [16], reflecting the denitrification process in the filter. In addition, *Flavobacterium psychrophilum*, belonging to the Flavobacteriaceae, has been found to be a harmful microorganism. *Flavobacterium psychrophilum* is a gram-negative, filamentous, psychrotrophic bacterium, and is the causative agent of bacterial cold-water disease and rainbow trout syndrome in freshwater salmonid fish worldwide, generating injuries and high mortality rates [17]. Therefore, more attention should be paid to this genus needs in the future. *Haloferula* belongs to the Chloroflexi and is also a facultative anaerobic microorganism. This taxon performs photosynthesis that neither produces oxygen nor fixes nitrogen [14]. Ammonia-oxidizing bacteria (AOB; nitrite bacteria) and nitrite-oxidizing bacteria (NOB; nitrite bacteria) performed nitrification in the biological filter in this study. Nitrifying and denitrifying microorganisms can greatly shorten the nitrogen removal time in biological filters and improve nitrogen removal efficiency [18–21]. *Nitrosomonas* was the main nitrifying bacteria in the treatment group. *Lactobacillus*, a beneficial microorganism [22], was detected in water samples from the outlet of the copper ion generator. *Methyloceanibacter* and *Mesorhizobium* were detected in the filter. Some studies [23] have found that adding Rhizobium to the fodder of *Litopenaeus vannamei* reduced the feed coefficient and improved shrimp growth, survival rate, body condition, specific growth rate, antioxidant enzyme activity, and disease resistance.

The results showed that the relative abundances of *Erythrobacter*, nitrite bacteria, and *Flavanobacteria* were higher in the treatment group than in the control group. These microorganisms were also the most abundant in the sewage treatment and nitrogen removal systems in the biofilm reactor. In addition, *Nitrosomonas* was the main nitrifying bacteria. Lactobacillus was detected in the water samples from the outlet of the copper ion generator. *Methyloceanibacter* and *Mesorhizobium* were detected in the filter. These microorganisms improve the environmental quality of aquaculture water and provide a foundation for efficient, high-yield aquaculture [24].

However, *norank_f_caldilineaceae* was detected in the filter of the control group. This taxon [25] is not conducive to the purification of aquaculture water and sometimes contains opportunistic pathogens and other commercially detrimental organisms in RASs which is similar to the results of Bartelme et al. [13].

Copper ion sterilization indirectly enriched the beneficial bacteria, which supported the production of a variety of digestive enzymes in a specific environment, promoted the digestion and absorption of nutrients, and improved the feed utilization rate.

Above all, in addition to characterizing the numbers and species of microorganisms in samples, it is important to collect data on their physiological states [26]. The method of bacterial community analysis used in this study provided only limited information on the activities and physiological states of microorganisms. It is necessary to combine metagenomic, single-cell microbiology to supplement and improve this study. For example, viability PCR could be used in future research to observe the activity levels of Rhodobacteraceae, Flavobacteriaceae, Haloferula, and nitrite bacteria.

In addition, the phenotypes and genotypes of microorganisms respond to different physicochemical stressors, including germicidal UV light and antimicrobials. Muñoz et al. studied the resistance, phenotype, and molecular response of *Lactobacillus* to different physicochemical stressors and found that the phenotypic response to stress was the same, but the induced and suppressed gene pools were different [27]. However, there were differences in the microflora genera between the treatment group and the control group in this study, especially with *Rhodobacteraceae*, *Flavobacteriaceae*, *Haloferula*, and nitrite bacteria. In addition, *Lactobacillus* was detected only in the water samples from the outlet of the copper ion generator. Therefore, correlating phenotypic and genotypic responses will provide new insights on how bacteria respond to a changing environment in the future.

## Conclusion

There was no significant enrichment of *B. rerio* when the copper ion concentration was 0.15 mg/L. In addition, microbiological analysis clearly showed that bacterial species richness and community diversity differed between the two groups. However, there was no sharp decrease in the microflora near the outlet of the copper ion generator. The relative abundances of *Erythrobacter*, nitrite bacteria, and *Flavanobacteria* were clearly higher in the treatment group than in the control group. Therefore, copper ion sterilization can be considered a relatively mild cleaning technology. Copper ion sterilization may greatly improve the abundance of beneficial bacteria, which may help control the organic matter and inorganic nitrogen pollution in RASs. These functional bacteria could be isolated and developed into bacterial agents for use in RASs in the near future. These bacterial agents have important prospective applications in the optimization of aquaculture processes, the improvement of aquaculture production, and the effective control of aquaculture disease risk. Incorporating this knowledge would provide opportunities to develop new system operations and could move system optimization boundary set the bound by current models. This would be expected to become popular in the aquaculture industry.

## Supplementary Information

Below is the link to the electronic supplementary material.

Supplementary file1 (docx 470 KB)

Supplementary file2 (docx 15 KB)

Supplementary file3 (docx 62 KB)

Supplementary file4 (pdf 209 KB)

Supplementary file5 (docx 14 KB)

Supplementary file6 (doc 148 KB)

Supplementary file7 (docx 48 KB)

## Data Availability

Data are available from the corresponding authors upon reasonable request.
