# Supplementary material for: Effect of Copper Ion Sterilization on Bacterial Community in a Freshwater Recirculating Aquaculture System
Source: Curr Microbiol. 2022 Jan 4;79(2):58. doi: 10.1007/s00284-021-02707-2 (PMC8727413; doi:10.1007/s00284-021-02707-2)
Supplement: Supplementary file 1 — Supplementary file1 (docx 470 KB) [file 284_2021_2707_MOESM1_ESM.docx]

**Fig. 1** the results of water quality. *In this figure, the treatment groups were marked T- and the control groups were marked C-.The concentrations of TAN, nitrate, and nitrite were 0.13 ± 0.08 mg/l, 0.02 ± 0.01 mg/l, and 0.02 ± 0.01 mg/l in the test group, 0.14 ± 0.07 mg/l, 0.05 + 0.04 mg/l, and 0.03 ± 0.02 mg/l in the control group.


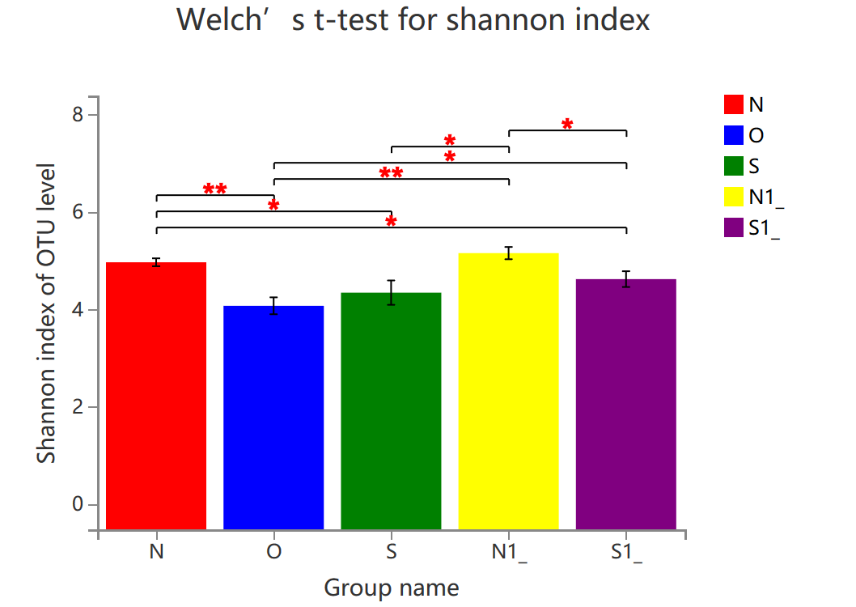


**Fig.** **2** Microbial diversity among groups, as measured by Shannon index*Data are expressed as mean ± standard deviation (S.D.) for each group (n = 3). *P < 0.05, **P < 0.01, ***P < 0.001. Error bars show the standard error of mean (S.E.M.), and the P-values are from two-tailed Student’s *t*-test.


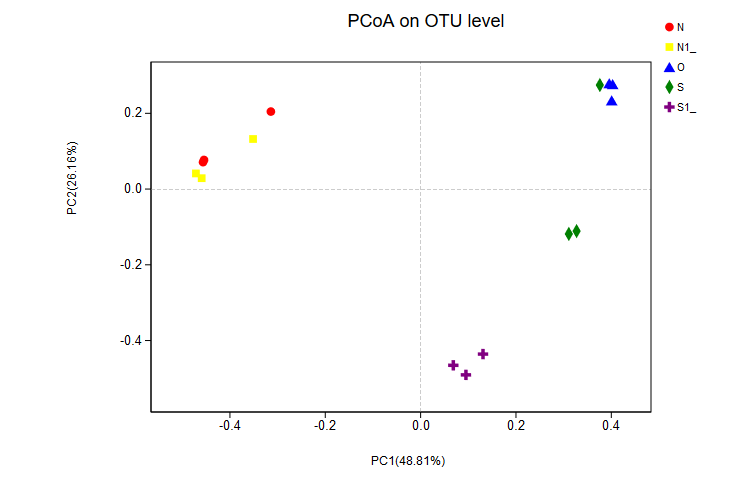


**Fig. 3** Principal coordinate analysis plots. *showing the microbiota in the treatment groups and the control groups (n = 3 per group) Principal co-ordinates analysis (PCoA) of the sequencing data identified differences between the treatment-group clusters and the control-group clusters, with the following main principal component (PC) scores: PC1 was 48.81%, and PC2 was 26.16%.

**Fig. 4** Analysis of Bacterial community composition in the filter and water samples on the phylum level *The five most-abundant bacterial classes in the control group were Actinobacteria (20.04%), Gammaproteobacteria (17.88%), Alphaproteobacteria (14.82%), Caldilineae (8.14%), and Deltaproteobacteria (4.86%), while the five most-abundant bacterial classes in the treatment group were Alphaproteobacteria (26.65%), Actinobacteria (13.34%), Flavobacteria (11.59%), Gammaproteobacteria (9.0%), and Thermomicrobia (8.81%).


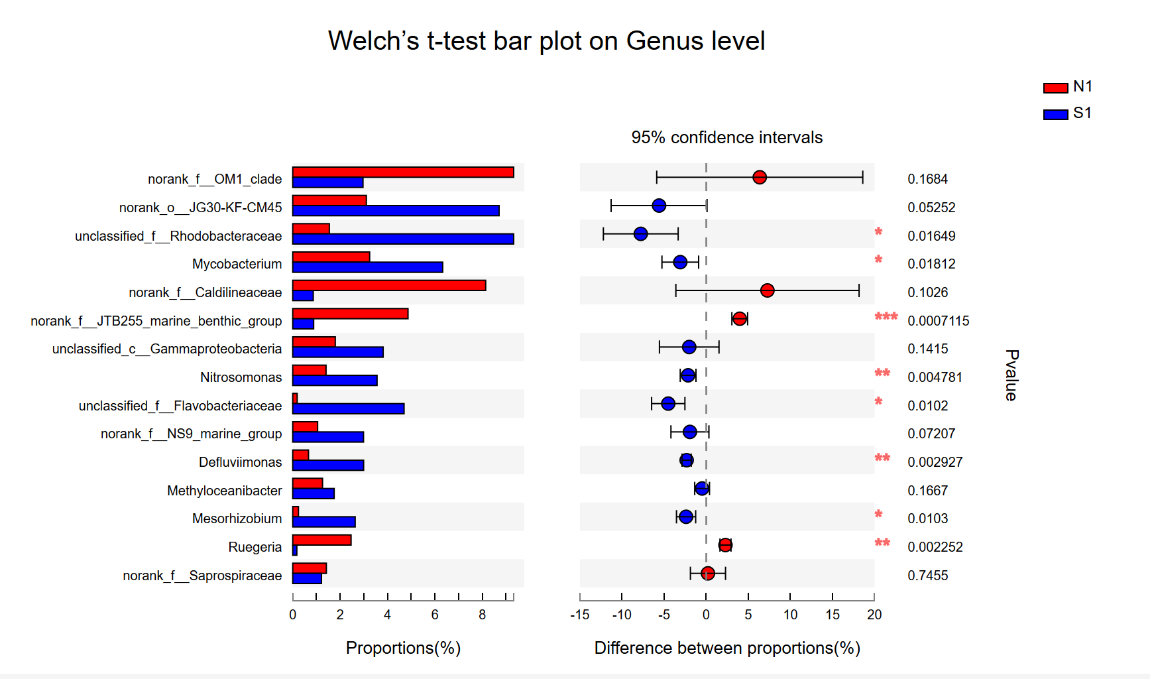
a


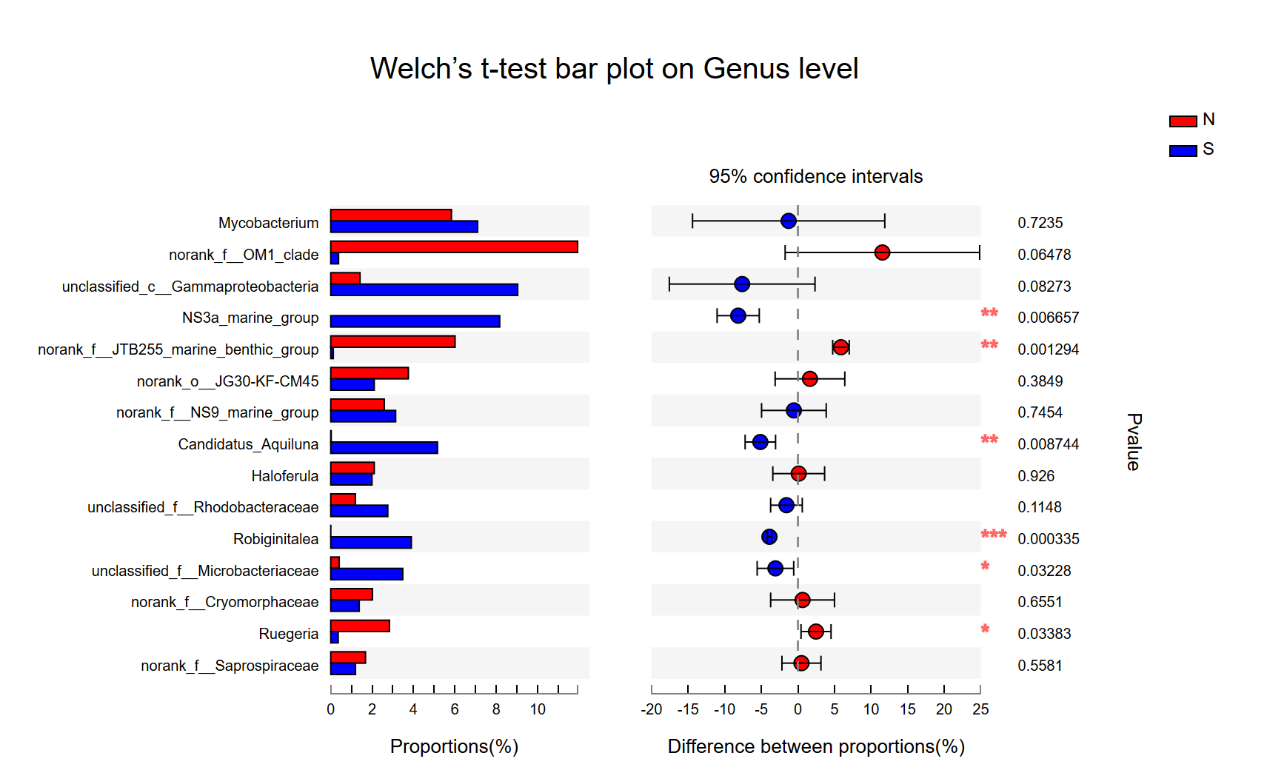
b


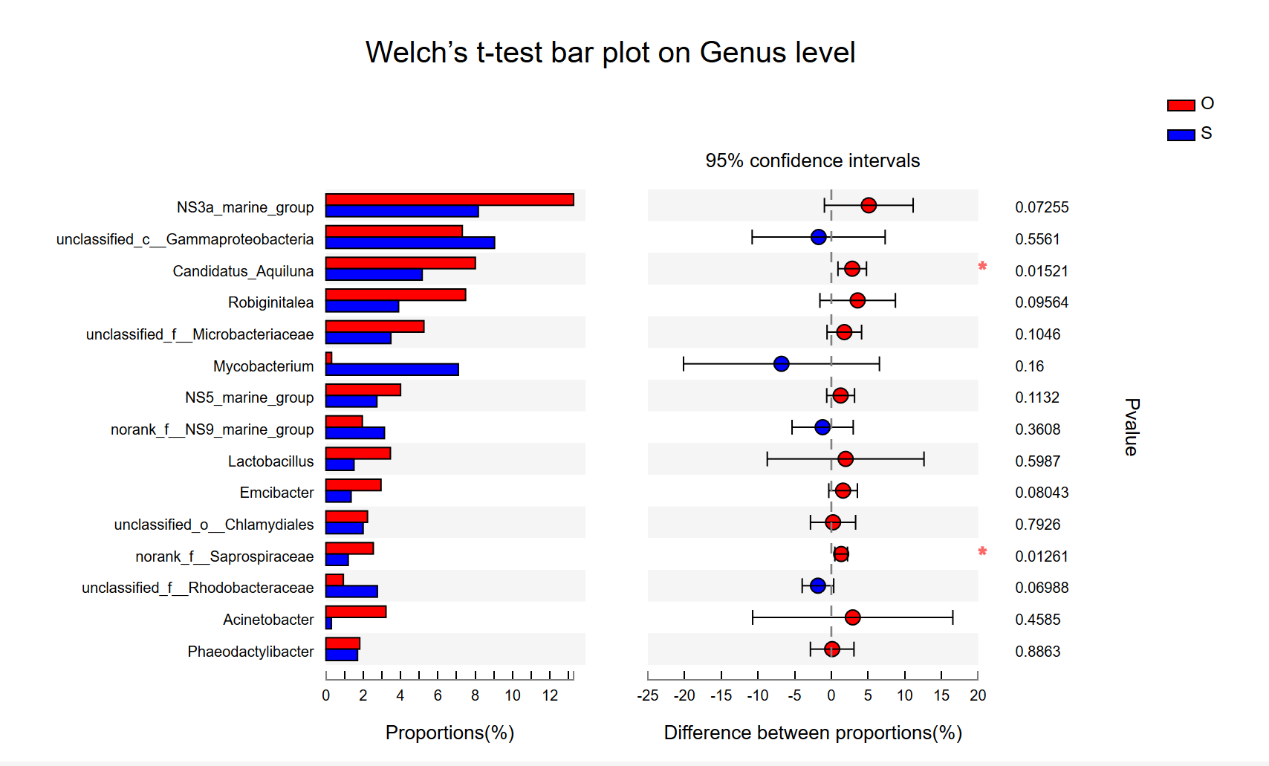
c

**Fig. 5** Significant differences in bacterial community composition between the treatment group and the control groups at the genus level*Statistical analysis was performed using Welch's *t* test (n = 3 per group). *P < 0.05, **P < 0.001; treatment group vs. control group.
