# Supplementary material for: Effect of Copper Ion Sterilization on Bacterial Community in a Freshwater Recirculating Aquaculture System
Source: Curr Microbiol. 2022 Jan 4;79(2):58. doi: 10.1007/s00284-021-02707-2 (PMC8727413; doi:10.1007/s00284-021-02707-2)
Supplement: Supplementary file 2 — Supplementary file2 (docx 15 KB) [file 284_2021_2707_MOESM2_ESM.docx]

| Table 1 Alpha-diversity data for the samples generated by high-throughput sequencing. | | | | | |
| --- | --- | --- | --- | --- | --- |
| Estimators | S | S1 | N1 | O | N |
| Shannon | 4.34±0.25 | 4.62 ± 0.16 | 5.15 ± 0.13 | 4.07 ± 0.17 | 4.96 ± 0.08 |
| Simpson | 0.03 ± 0 | 0.03 ± 0 | 0.02 ± 0 | 0.04 ± 0.01 | 0.02 ± 0 |
| Chao | 536.25 ± 355.64 | 739.72 ± 23.66 | 981.11 ± 75.97 | 403.24 ± 144.49 | 803.89 ± 453.28 |
| Coverage | 0.99 ± 0 | 0.99 ± 0 | 0.99 ± 0 | 1.00 ± 0 | 0.99 ± 0 |

^†^Data are expressed as mean ± standard deviation (S.D.) for each group (n = 3).
