# Supplementary material for: Effect of Copper Ion Sterilization on Bacterial Community in a Freshwater Recirculating Aquaculture System
Source: Curr Microbiol. 2022 Jan 4;79(2):58. doi: 10.1007/s00284-021-02707-2 (PMC8727413; doi:10.1007/s00284-021-02707-2)
Supplement: Supplementary file 3 — Supplementary file3 (docx 62 KB) [file 284_2021_2707_MOESM3_ESM.docx]

**Effect of copper ion sterilization on bacterial community in a freshwater** **recirculating aquaculture system**

**Acknowledgments** This work was supported by funds from the National Key Research and Development Project of China (No. 2019YFD0900501) and the China Agriculture Research System (CARS-47-G20).

**Abstract**

The study aimed to evaluate the safety of copper ion sterilization based on copper ion residues in zebrafish (*Brachydanio rerio*), as well as bacterial community structure and diversity in recirculating aquaculture systems (RASs). The copper ion content was determined using food safety standard GB 5009.13-2017. Bacterial community structures and alpha and beta diversity indexes were examined using the 16S rRNA gene sequences produced by Illumina HiSeq sequencing. The results revealed no significant copper ion enrichment in *B. rerio* when the copper ion concentration was 0.15 mg/L. The relative abundances of *Erythrobacter*, nitrite bacteria, and *Flavanobacteria* were clearly higher in the treatment group than in the control, and differences in bacterial species richness and diversity were obvious. In addition, there was no sharp decrease in the microflora at the outflow of the copper ion generator. In conjunction with the changes in ammonia nitrogen, nitrate, and nitrite concentrations during the experiment, the results indicated that there were no significant effects on the purification efficacy of the biological filter, but the abundances of beneficial bacteria increased significantly. This is of great relevance in order to understand the response of bacterial communities affected by changing environmental conditions such as copper ion sterilization.

**Keywords**: RAS·copper ion sterilization technology·high-throughput DNA sequencing·bacterial community

**Introduction**

As the aquaculture industry has developed, the number of farmed aquatic species has increased [1]. In addition, improved living standards have allowed consumers to focus on comprehensive nutrition and to demand increasingly higher safety standards in aquaculture production [2]. The health of the aquaculture environment is closely related to the microbial community structure in the water, as well as the levels of nutrients [3]. Microbial community structure, which reflects the biochemical reactions in the ecosystem, can be used as an indicator of the ecological health of the aquaculture environment [4]. Because aquaculture species must be fed constantly, native microorganisms cannot decompose organic matter rapidly enough to prevent water quality deterioration and the growth of harmful microorganisms [5]. The excess undecomposed feed not only pollutes the aquatic environment but also inhibits the respiration of other aquatic animals, possibly even leading to death [6]. High levels of organic matter pollution may also be toxic to humans [7]. However, beneficial microorganisms may play a role in water purification and may serve as food for aquatic organisms [8–9]. Therefore, beneficial and harmful aquatic bacteria should be analyzed effectively to ensure the health of aquaculture environments.

Sterilization technology can effectively mitigate the conflict between ecological benefits and economic interests, supporting sustainable development [10]. Copper ion sterilization is an electrochemical sterilization method, and copper ions are also one of the most common heavy metal contaminants in water [7]. The author's previous study found that the concentrations of nitrite, nitrate, and total ammonia nitrogen (TAN) and the total number of bacteria remained in a stable state when the concentration of Cu^2+^ was 0.1–0.2 mg/L and the breeding temperature was (26±2)°C. These conditions were beneficial for the growth of zebrafish (*Brachydanio rerio*) [7]. However, it is unclear whether *B. rerio* retains copper ions over the long term in aquaculture.

This study thoroughly discusses the safety of the copper ion sterilization process in aquaculture water, and provides a theoretical reference for the aquaculture industry with respect to copper ion residue and the microbiome.

**Materials and Methods**

The experimental system included a storage tank, a biochemical cotton filter, a moving bed biofilm reactor (MBBR), and a copper ion generator. The filter media parameters were as follows: K5, Ф25*4 mm, 64 holes, the specific surface area was 800 m^2^/m^3^, the bulk number was 2.1*10^5^ per m^3^, the bulk weight was 125 kg/m^3^, and the void ratio was 85%. The copper ion generator used was a YLD-TLZ-25, Jiangsu Yilida Water Treatment Systems Co., Ltd., China.

Two recirculating aquaculture systems (RASs) for *B. rerio* were constructed. *B. rerio* were acclimated for seven days in an RAS before the experiment began. Fifty healthy *B. rerio* with an average length of 2.5–3.0 cm and a weight of 0.30±0.05 g were selected.

The fish were divided into a control group and a treatment group. The control and treatment groups were exposed to 0 mg/L and 0.15 mg/L copper ions, respectively. Daily management operations included regular feeding and system sewage treatment. The copper ion concentration was tested three times per week, while TAN, nitrite, nitrate, dissolved oxygen (DO), temperature (T), and pH were tested daily. Samples of the water and filter material were collected at the end of the experiment. TAN, nitrate, and nitrite concentrations were determined using Nessler’s reagent spectrophotometry, zinc-cadmium reduction, and diazotization coupling, respectively. This experiment was performed at the Fisheries Equipment and Engineering research base of the Fishery Machinery and Instrument Research Institute, Chinese Academy of Fisheries Sciences, China in July 2018. The experiment lasted one month.

The RAS water samples (100 mL each) and the filter media samples from the MBBR (100 g each) were collected in sterile tubes separately and transferred to the laboratory. Samples were collected in triplicate. The filter was washed with sterile fresh water and shaken vigorously to dissolve the biofilm on the carrier. Then, the above solutions were filtered onto 0.22-μm filters and frozen at −80°C. Ten *B. rerio* each in the treatment group and control group were randomly selected to measure the amount of the copper ion residues.

In the treatment group, the biological filter sample was marked S1, the water sample was marked S, and the water sample from the outlet of the copper ion generator exit was marked O. In the control group, the biological filter sample was marked N1, and the water sample was marked N.

To maximize read depth for a temporal study of the bacterial communities in the RAS, we used the Illumina HiSeq platform and targeted the V3–V4 region of the 16S rRNA gene. The V3–V4 region of the bacterial 16S rRNA gene was amplified using the forward and reverse universal primers 338F (5’-ACTCCTACGGGAGGCAGCA-3’) and 806R (5’-GGACTACHVGGGTTCTAAT-3’), respectively. The amplicon mixture was applied to a HiSeq 2500 Genome Sequencer (Illumina, San Diego, CA, USA). Bacterial community composition and biodiversity were determined by Shanghai Majorbio Bio-Pharm Technology Co., Ltd. (Shanghai, China).

**Data analysis**

The extracted high-quality sequences were aligned using PyNAST and UCLUST. The unique sequences were classified into operational taxonomic units (OTUs) at a threshold of 97% identity using UCLUST. Chimera Slayer was used to remove potential chimeric sequences from the set of representative OTUs. MOTHUR was used for data analysis. The data were analyzed on the free online platform of Majorbio Cloud Platform (www.majorbio.com). Statistical analyses were performed using SPSS V.17.0 [11]. The mean (M) and standard deviation (SD) were used to explore the relationship between specific explanatory variables and outcome variables for water quality results and the concentration of copper ions in *B. rerio* after exposure to 0.15 mg/L copper ions.

**NCBI sequence accession numbers**

Bacterial V3-V4 16S rRNA gene sequences generated in this study are available from the NCBI SRA (SRP297447).

**Results**

**RAS water quality parameters**

The concentrations of TAN, nitrate, and nitrite in the test group were 0.04–0.35 mg/L, 0.04–0.01 mg/L, and 0.03–0.01 mg/L, respectively, with averages of 0.13 ± 0.08 mg/L, 0.02 ± 0.01 mg/L, and 0.02 ± 0.01 mg/L, respectively. In the control group, the concentrations of TAN, nitrate, and nitrite were 0.05–0.26 mg/L, 0.18–0.01 mg/L, and 0.08–0.02 mg/L, respectively, with averages of 0.14 ± 0.07 mg/L, 0.05 + 0.04 mg/L, and 0.03 ± 0.02 mg/L, respectively (**Fig. 1**).

**Residual** **copper ions in *B. rerio***

*B. rerio* were sampled one day after the last feeding. The copper ion content was determined using GB 5009.13-2017 (NHC 2017). The average values of the cupric ion concentrations in both the treatment and control groups were 16.3 mg/kg.

**Influence of copper ions on the microbiology of the RAS**

Based on homologous sequence alignments and clustering, using the information extracted from the RDP and BLAST databases, the OTUs were taxonomically identified to the lowest level possible. The Shannon diversity curves for those samples reached the saturation phase, indicating that the majority of the bacterial phylotypes in the sample had been identified (**Fig. 2**).

Alpha-diversity data for the samples generated by high-throughput sequencing are shown in **Table 1**, including the Shannon, Simpson, Chao, and Coverage indexes. The estimated sample coverage (Good’s coverage) was about 99%, which indicated that sequencing accuracy and reprehensibility was acceptable. No significant difference in estimated OTU richness (Chao) was observed between the two groups.

However, significant differences in estimations of community diversity between the two groups were identified, including the Shannon index at 4.62 ± 0.16 in the control vs. 5.15 ± 0.13 in the treatment group, with P = 0.01, and 4.34 ± 0.25 in the control vs. 4.96 ± 0.08 in the treatment group, with P = 0.04 for the filter and water samples, respectively, indicating that microbiota diversity was greater in the treatment groups. In addition, there were no significant differences in estimations of community diversity between the S and the O in the treatment groups (Shannon 4.34 ± 0.25 vs. 4.07 ± 0.17, respectively, P = 0.2) (**Table 1** and **Fig. 3**)

Bacterial differences were further investigated. Principal co-ordinates analysis (PCoA) of the sequencing data identified differences between the treatment-group clusters and the control-group clusters, with the following main principal component (PC) scores: PC1 was 48.81%, and PC2 was 26.16%. These PC scores demonstrated that the clustering pattern differed among groups (**Fig. 4**). N and N1 were in the same area and were distinct from S, S1, and O, which indicated that bacterial community structure differed between the treatment group and the control group. This suggested that copper ion sterilization had a significant effect in the RAC.

The species abundance of each sample was calculated at different taxonomic levels by community composition analysis. At the phylum level, the relative abundance of the Proteobacteria was high in filter material from both the control group and the treatment group, accounting for 31.6% and 42.4%, respectively (**Fig. 5**). The five most abundant bacterial classes in the control group were Actinobacteria (20.04%), Gammaproteobacteria (17.88%), Alphaproteobacteria (14.82%), Caldilineae (8.14%), and Deltaproteobacteria (4.86%), while the five most abundant bacterial classes in the treatment group were Alphaproteobacteria (26.65%), Actinobacteria (13.34%), Flavobacteria (11.59%), Gammaproteobacteria (9.0%), and Thermomicrobia (8.81%).

There were differences in the water samples between the treatment group and the control group. The dominant phylum in the control group was Actinomycetes, accounting for 40.10%, while the dominant phylum in the treatment group was Bacteroidetes, accounting for 38.90% in the S and 48.80% in the O (**Fig. 5**). However, the communities of low-abundance microbes were similar between the S and O in the treatment group, indicating that the aquatic environment was homogeneous.

Species difference analysis revealed significant differences between groups by Welch’s t test. There were differences in the microflora genera between the treatment group and the control group. The genera that were significantly different in the filter included *Mycobacterium*, *Rhodobacteraceae*, *Defluviimonas*, *Flarobacterium*, *Nitrosomonas*, *Mesorhizobium*, and *Ruegeria*, and the overwhelmingly dominant genus was *norank_f__JTB255_marine_benthic_group*. However, in water, *NS3a_marine_group*, *norank_f__JTB255_marine_benthic_group*, *Candidatus_Aquiluna*, *unclassified_f__Microbacteriaceae*, and *Ruegeria* were significantly different, and the overwhelmingly dominant genus was *Robiginitalea*. Both *norank_f__JTB255_marine_benthic_group* and *Ruegeria* were more abundant in the control group than in the treatment group. In addition, there were significant differences in the abundance of the same strain between the water samples and filter materials (**Fig. 6**).

In the treatment group, only *Candidatus_Aquiluna* and *norank_f__Saprospiraceae* were significantly different between the S and O. In the control group, *norank_f__Caldilineaceae* and *norank_f__Saprospiraceae* were detected on the filter, while *norank_f__Saprospiraceae*, *Haloferula* and *norank_f__Cryomorphaceae* were detected in the water (**Fig. 6**).

The relative abundances of *Erythrobacter*, nitrite bacteria*,* and *Flavanobacteria* were greater in the treatment group than in the control group. These microorganism were also most abundant in the sewage treatment and nitrogen removal systems in the biofilm reactor.

**Discussion**

Various factors affect the survival of aquatic economic animals in RASs, including differences in system scale, water properties, and microbial community composition.

Suitable water conditions are essential for the survival of aquatic economic animals such as fish. In this experiment, there were no obvious changes in TAN, nitrate, and nitrite when the copper ions were 0.15 mg/L. The average values of the tested water properties did not reach harmful levels that would negatively impact aquaculture organisms [7]. In addition, there was no significant enrichment of copper ions in *B. rerio* when the copper ions were 0.15 mg/L.

Microbial ecological theory in aquaculture has the potential to extend RAS capabilities [12]. Further identifying the interactions between microorganisms and system design could alleviate concerns regarding the sustainability of aquaculture. The results of Bartelme et al. [13] studied the bacterial and archaeal community structure of a commercial-scale freshwater RAS raising *Perca flavescens* (yellow perch) and found that >99.9% of the archaeal 16S rRNA gene sequences were classified to a single taxon associated with known ammonia-oxidizing archaea. Therefore, archaea will be focused on in a spatial study related to nitrification in the future.

It is likely true that each microbial community assemblage will be unique among RASs, i.e., each RAS has a unique “microbial fingerprint” [13]. The Shannon index, Simpson diversity index, Chao1, and observed species in each sample were used to evaluate species richness and diversity. The results indicated that bacterial species richness and community diversity varied distinctly among the samples.

There were differences in the microflora genera between the treatment group and the control group, especially within *Rhodobacteraceae, Flavobacteriaceae, Haloferula*, and nitrite bacteria*.* *Rhodobacteraceae unclassified* belongs to the family *Rhodobacteraceae*, in the Alphaproteobacteria, and is a purple non-sulfur bacteria [14]. Depending on extracellular enzymes, heterotrophic metabolic reactions can be carried out by utilizing a variety of organic carbon sources in aquaculture water [14]. In anaerobic or hypoxic conditions, *Rhodobacteraceae* effectively absorbs phosphate, reduces the phosphorus load in high-density aquaculture, and can play a role in reducing the chemical oxygen demand of water [15]. *Flav**obacteriaceae-*unclassified, which belongs to the *Flavobacteriaceae* (Bacteroidetes), is a facultative anaerobic bacterium. This bacterium performs dissimilatory nitrate reduction [16], reflecting the denitrification process in the filter. In addition, *Flavobacterium psychrophilum*, belonging to the Flavobacteriaceae, has been found to be a harmful microorganism. *Flavobacterium psychrophilum* is a gram-negative, filamentous, psychrotrophic bacterium, and is the causative agent of bacterial cold-water disease and rainbow trout syndrome in freshwater salmonid fish worldwide, generating injuries and high mortality rates [17]. Therefore, more attention should be paid to this genus needs in the future. *Haloferula* belongs to the Chloroflexi, and is also a facultative anaerobic microorganism. This taxon performs photosynthesis that neither produces oxygen nor fixes nitrogen [14]. Ammonia-oxidizing bacteria (AOB; nitrite bacteria) and nitrite-oxidizing bacteria (NOB; nitrite bacteria) performed nitrification in the biological filter in this study. Nitrifying and denitrifying microorganisms can greatly shorten the nitrogen removal time in biological filters and improve nitrogen removal efficiency [18–21]. *Nitrosomonas* was the main nitrifying bacteria in the treatment group. *Lactobacillus*, a beneficial microorganism [22], was detected in water samples from the outlet of the copper ion generator. *Methyloceanibacter* and *Mesorhizobium* were detected in the filter. Some studies [23] have found that adding Rhizobium to the fodder of *Litopenaeus vannamei* reduced the feed coefficient and improved shrimp growth, survival rate, fatness, specific growth rate, antioxidant enzyme activity, and disease resistance.

The results showed that the relative abundances of *Erythrobacter*, nitrite bacteria, and *Flavanobacteria* were higher in the treatment group than in the control group. These microorganisms were also the most abundant in the sewage treatment and nitrogen removal systems in the biofilm reactor. In addition, *Nitrosomonas* was the main nitrifying bacteria. *Lactobacillus* was detected in the water samples from the outlet of the copper ion generator. *Methyloceanibacter* and *Mesorhizobium* were detected in the filter. These microorganisms improve the environmental quality of aquaculture water, and provide a foundation for efficient, high-yield aquaculture [24].

However, *norank_f_caldilineaceae* was detected in the filter of the control group. This taxon [25] is not conducive to the purification of aquaculture water and sometimes contains opportunistic pathogens and other commercially detrimental organisms in RASs, which is similar to the results of Bartelme, R. P. et al. [13].

Copper ion sterilization indirectly enriched the beneficial bacteria, which supported the production of a variety of digestive enzymes in a specific environment, promoted the digestion and absorption of nutrients, and improved the feed utilization rate.

Above all, in addition to characterizing the numbers and species of microorganisms in samples, it is important to collect data on their physiological states [26]. The method of bacterial community analysis used in this study provided only limited information on the activities and physiological states of microorganisms. It is necessary to combine metagenomic, single-cell microbiology to supplement and improve this study. For example, viability PCR could be used in future research to observe the activity levels of *Rhodobacteraceae, Flavobacteriaceae, Haloferula*, and nitrite bacteria.

In addition, the phenotypes and genotypes of microorganisms respond to different physicochemical stressors, including germicidal UV light and antimicrobials. Casado Muñoz et al. studied the resistance, phenotype, and molecular response of lactic acid bacteria to different physicochemical stressors and found that the phenotypic response to stress was the same, but the induced and suppressed gene pools were different [27]. However, there were differences in the microflora genera between the treatment group and the control group in this study, especially in *Rhodobacteraceae*, *Flavobacteriaceae*, *Haloferula*, and nitrite bacteria. In addition, *Lactobacillus* was detected only in the water samples from the outlet of the copper ion generator. Therefore, correlating phenotypic and genotypic responses will provide new insights on how bacteria respond to a changing environment in the future.

**Conclusion**

There was no significant enrichment of *B. rerio* when the copper ion concentration was 0.15 mg/L. In addition, microbiological analysis clearly showed that bacterial species richness and community diversity differed between the two groups. However, there was no sharp decrease in the microflora near the outlet of the copper ion generator. The relative abundances of *Erythrobacter*, nitrite bacteria, and *Flavanobacteria* were clearly higher in the treatment group than in the control group. Therefore, copper ion sterilization can be considered a relatively mild cleaning technology. Copper ion sterilization may greatly improve the abundance of beneficial bacteria, which may help control the organic matter and inorganic nitrogen pollution in RASs. These functional bacteria could be isolated and developed into bacterial agents for use in RASs in the near future. These bacterial agents have important prospective applications in the optimization of aquaculture processes, the improvement of aquaculture production, and the effective control of aquaculture disease risk. Incorporating this knowledge would provide opportunities to develop new system operations, and could move system optimization beyond the boundary set by current models. This would be expected to become popular in the aquaculture industry.

**Compliance with ethical standards**

The authors declare that they have no conflicts of interest and data availability.

**Author contributions statement**

All authors contributed to the study conception and design. Material preparation was performed by Shi Chen and Chongwu Guan, and data collection and analysis were performed by Jianjun Shan and Chongwu Guan. The first draft of the manuscript was written by Jianjun Shan and Xiaoqing Tian. Yulei Zhang and Chenglin Zhang contributed to the comments for manuscript edits. All authors commented, read, consented to participate, consented to publication, and approved the final manuscript.

Figure legends

| No. | Figure caption |
| --- | --- |
| Fig. 1 | Water quality results |
| Fig. 2 | Shannon diversity curves for the samples |
| Fig. 3 | Microbial diversity among groups, as measured by Shannon index, Simpson diversity index, and Chao1 |
| Fig. 4 | Principal coordinate analysis plots, showing the microbiota in the treatment groups and the control groups |
| Fig. 5 | Bacterial community composition in the filter and water samples at the phylum level |
| Fig. 6 | Significant differences in bacterial community composition between the treatment group and the control groups at the genus level |

Table 1 Alpha-diversity data for the samples generated by high-throughput sequencing
