# Supplementary material for: Effect of Copper Ion Sterilization on Bacterial Community in a Freshwater Recirculating Aquaculture System
Source: Curr Microbiol. 2022 Jan 4;79(2):58. doi: 10.1007/s00284-021-02707-2 (PMC8727413; doi:10.1007/s00284-021-02707-2)
Supplement: Supplementary file 4 — Supplementary file4 (pdf 209 KB) [file 284_2021_2707_MOESM4_ESM.pdf]

# Certificate of English Language Editing

## Manuscript Title:

Effect of copper ion sterilization on bacterial community in a freshwater recirculating aquaculture system

## Date of Revision:

October 18, 2021

Abstract:

The study aimed to evaluate the safety of copper ion sterilization based on copper ion residues in zebrafish (Brachydanio rerio), as well as bacterial community structure and diversity in recirculating aquaculture systems (RASs). The copper ion content was determined using food safety standard GB 5009.13-2017. Bacterial community structures and alpha and beta diversity indexes were examined using the 16S rRNA gene sequences produced by Illumina HiSeq sequencing. The results revealed no significant copper ion enrichment in B. rerio when the copper ion concentration was 0.15 mg/L. The relative abundances of Erythrobacter, nitrite bacteria, and Flavanobacteria were clearly higher in the treatment group than in the control, and differences in bacterial species richness and diversity were obvious. In addition, there was no sharp decrease in the microflora at the outflow of the copper ion generator. In conjunction with the changes in ammonia nitrogen, nitrate, and nitrite concentrations during the experiment, the results indicated that there were no significant effects on the purification efficacy of the biological filter, but the abundances of beneficial bacteria increased significantly. This is of great relevance in order to understand the response of bacterial...

This document certifies that the manuscript listed above was copy edited for English language by LetPub, with regard to grammar, punctuation, spelling, and clarity. All of our language editors are native English speakers with long-term experience in editing scientific and technical manuscripts. We are committed to leveling the playing field for researchers whose native language is not English.

- Documents receiving this certification should be regarded as having undergone professional editorial revision for English language before submission. However, the authors may accept or reject LetPub's suggestions and changes at their own discretion and LetPub does not have editorial control over the submitted documents.
- The language quality of the submitted document is the sole responsibility of the submitting authors subject to those authors' adherence to LetPub's revisions and instruction. LetPub's provision of service does not constitute a guarantee or endorsement of the authors' work herein.
- Neither the research content nor the authors' intended meaning were altered in any way during the editing process.
- If you have any questions or concerns about this edited document, please contact us at support@letpub.com

LetPub is an author service brand owned and operated by Accdon LLC. Headquartered in the Boston area, we are a full-spectrum author services company with a large team of US-based certified language and scientific editors, ISO 17001 accredited translators, and professional scientific illustrators and animators. We advocate ethical publication practices and are an official member of the Committee on Publication Ethics (COPE).

For more information about our company, services, and partnership programs, please visit www.letpub.com.

© 2021 Accdon, LLC. All Rights Reserved. Tel: 1-781-202-9968 Email: info@accdon.com Address: 400 Fifth Ave, Suite 530, Waltham, MA 02451, United States
