# Supplementary material for: Effect of Copper Ion Sterilization on Bacterial Community in a Freshwater Recirculating Aquaculture System
Source: Curr Microbiol. 2022 Jan 4;79(2):58. doi: 10.1007/s00284-021-02707-2 (PMC8727413; doi:10.1007/s00284-021-02707-2)
Supplement: Supplementary file 7 — Supplementary file7 (docx 48 KB) [file 284_2021_2707_MOESM7_ESM.docx]

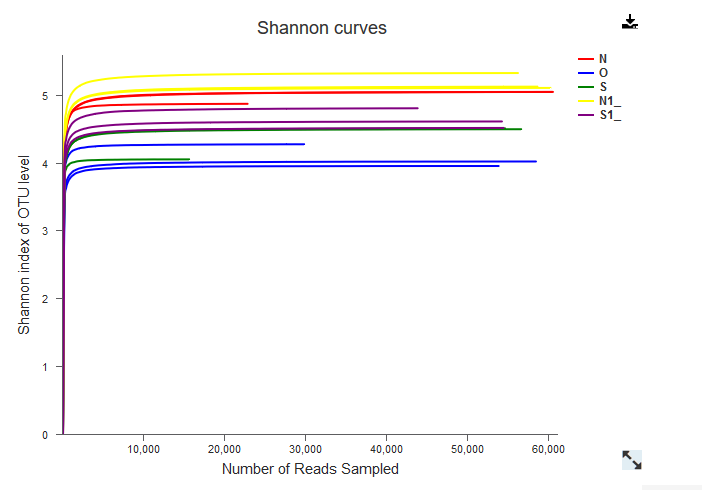


**Fig. I** the dilution curve based on Shannon index for the samples * The amount of sequencing data in each samples is sufficient according to the curve flattens out yet.
