# Supplementary material for: Effect of Copper Ion Sterilization on Bacterial Community in a Freshwater Recirculating Aquaculture System
Source: Curr Microbiol. 2022 Jan 4;79(2):58. doi: 10.1007/s00284-021-02707-2 (PMC8727413; doi:10.1007/s00284-021-02707-2)
Supplement: Supplementary file 5 — Supplementary file5 (docx 14 KB) [file 284_2021_2707_MOESM5_ESM.docx]

Dear Editors:

Thank you very much for the comments and suggestions. According to the comments of reviewers, we have provided a point-by-point response and resubmit it to the Journal of Current Microbiology. The comments and explanations are attached for you.

We deeply appreciate your consideration of our manuscript, and we look forward to receiving comments. If you have any queries, please don’t hesitate to contact me at the address below.

Thank you and best regards.

Yours sincerely,

Jianjun Shan
